# Plant Virus Expression Vectors: A Powerhouse for Global Health

**DOI:** 10.3390/biomedicines5030044

**Published:** 2017-07-30

**Authors:** Kathleen Hefferon

**Affiliations:** Department of Food Sciences, Cornell University, Ithaca, NY 14850, USA; klh22@cornell.edu; Tel.: +1-607-280-0094

**Keywords:** vaccine, virus expression vector, Tobacco mosaic virus, potato virus X, Cowpea mosaic virus, nanoparticle

## Abstract

Plant-made biopharmaceuticals have long been considered a promising technology for providing inexpensive and efficacious medicines for developing countries, as well as for combating pandemic infectious diseases and for use in personalized medicine. Plant virus expression vectors produce high levels of pharmaceutical proteins within a very short time period. Recently, plant viruses have been employed as nanoparticles for novel forms of cancer treatment. This review provides a glimpse into the development of plant virus expression systems both for pharmaceutical production as well as for immunotherapy.

## 1. Introduction

Plant-derived biopharmaceuticals, including vaccines, monoclonal antibodies, and other therapeutic proteins, are rapidly emerging into the marketplace. Today, commercialized plant-made pharmaceutical products are offered by Protalix Bio Therapeutics, Inc. (Karmiel, Israel), Planet Biotechnology, Inc. (Hayward, CA, USA), and Icon Genetics, GmbH (Halle, Germany). Biologics derived from plants have been demonstrated to be effective, safe, and inexpensive. They are easy to generate en masse and can be stored at ambient temperatures [1]. These attributes make plant-derived biopharmaceuticals attractive alternatives for providing medicines that were previously inaccessible to poor developing countries. While this longstanding goal has yet to be realized, plant-derived vaccines and other pharmaceuticals have found additional applications. For example, plant-made vaccines can be stockpiled as a measure of protection against global pandemics or biological warfare threats, and can be used for providing personalized medicines that are both rapid in turnaround and affordable to implement [2,3].

The first plant-derived pharmaceuticals were generated from transgenic plants, with length of generation time and protein expression levels being two of the principal caveats of this technological approach. In response, transient expression through the use of plant virus expression vectors became an attractive venue by which biopharmaceuticals could also be generated [4]. Virus vector-based expression systems could simultaneously shorten production time and increase pharmaceutical yield, while removing concerns from the general public about the use of genetically modified organisms (GMOs). More recently, plant viruses have found a niche in the field of cancer immunotherapy, by acting as nanoparticles which can home in on and elicit a form of highly localized immune response to solid tumors. Plant virus nanoparticles can even be engineered to carry a drug payload to cancer cells, thus offering a new and potent arsenal for cancer researchers and physicians alike [5].

With this history, the development of plant virus expression vectors for biopharmaceutical development has become a powerful tool. This review aims to describe some of the virus expression systems currently in use and explore some of their most recent applications, both as production systems and as nanoparticles for cancer immunotherapy.

## 2. Design of Plant Virus Expression Vectors

Plant viruses have been engineered to express vaccines, monoclonal antibodies, and other therapeutic proteins. Plant virus expression vectors have been designed from the genomes of both positive-sense RNA viruses or single-stranded DNA viruses [6,7,8]. First generation virus expression vectors are based on a full virus strategy and contain the entire virus genome, with the foreign gene of interest expressed from the same open reading frame as the coat protein, either as part of a fusion protein or else separately from an additional strong subgenomic promoter that is incorporated into the viral genome. Vaccine epitopes can be presented on the surface of virus particles by insertion into external portions of the virus capsid protein. The collective repeat pattern that results is better recognized by the immune system. The full-virus strategy presents several limitations, such as restrictions with respect to the size of the foreign protein that can be expressed.

Second generation virus expression vectors, on the other hand, have no size limitation of foreign genes, have improved production levels, and overcome both host plant species and tissue restrictions. These new ‘deconstructed vectors’ are composed solely of the foreign gene of interest and the minimum virus components that are required for replication [9,10,11]. As a consequence of the removal of genes essential to virus transport and assembly, for example, deconstructed vectors must be delivered to the host plant by alternative means, such as vacuum infiltration of the agrobacterium suspension that harbors the expression vector into plant leaves [12]. This synchronous production of the desired pharmaceutical protein in all plant tissues can increase protein production in a reduced time period.

A third plant virus strategy using viruses as nanoparticles has arisen as a means by which, among other things, cancer can be addressed. Immunotherapy using plant virus nanoparticles has been demonstrated to elicit a localized immune response against cancer cells that is also stable, nontoxic, and biodegradable [13]. The external surfaces of rod-shaped plant viruses, such as potexviruses, can be functionalized to target and deliver antiviral agents into tumors. Icosahedral plant viruses, such as the Cowpea mosaic virus, can be modified to carry cargo molecules, such as drugs, within their internal cavities. In this way, plant viruses can be used for diagnosis as well as for immunotherapy [14,15].

## 3. Vectors Based on Tobamoviruses

Rigid rod-shaped tobamoviruses include the Tobacco mosaic virus (TMV). TMV has 2130 copies of the capsid protein (CP), and was the first virus that has been engineered as a deconstructed vector system [10,11]. This new expression system is composed of two modules; one containing the replicase, the portion of the genome responsible for viral replication, and the other containing the site of foreign gene expression. TMV can be used to express both full length proteins and epitopes in the form of fusion proteins, and examples include Noris et al., Huang et al., Musichek et al., and Mett et al. [16,17,18,19]. Icon Genetics, a biotechnology company based in Germany, developed a technique for transfecting plants with these recombinant virus vector modules, known as Magnifection [11]. Magnifection combines agroinfiltration with the delivery of a deconstructed vector that lacks the ability to spread to other plants. A deconstructed TMV vector has been employed to generate Human papillomavirus HPV E7 protein and Norwalk virus-like particles (VLPs) in plants [20,21], as well as the Influenza M2e epitope in plants [16]. In addition, a “launch vector” based on a TMV that is housed within an *Agrobacterium tumefaciens* binary vector has also been developed by Musiychuk et al. in 2007 to generate vaccines against cholera, the influenza virus, and the plague [22,23,24,25].

Foreign protein expression in TMV infected plants can also be enhanced significantly through the coexpression of the RNA silencing suppressor gene P19 of the Tomato bushy stunt virus [26]. Other TMV-based vectors have been designed; for example, the TMV RNA-based overexpression (TRBO) vector can increase expression several times by removing the coat protein gene and placing the foreign gene open reading frame (ORF) closer to the 3′ end of the TMV RNA [27,28]. As another example, TMV has undergone extensive development by Fraunhofer USA as a potential vaccine against the pandemic H1N1 influenza virus, and clinical trials are currently underway [29,30,31,32]. Others have also examined the parameters of TMV-based influenza vaccine development. For example, Matsuda et al. found that temperature post viral vector inoculation influenced the spatial expression of hemagglutinin (HA) content in *Nicotiana benthamiana* leaves [33]. The authors determined that 20 °C is the optimal temperature required to obtain a maximal and stable yield of HA. Similarly, Patil et al. have shown that light intensity can affect the movement of viral gene products [34].

Liu and Kearney have developed a tobamovirus that infects legumes, based on Sun Hemp Mosaic Virus (SHMV) that incorporates some of the attributes of the strategies developed by Icon and TRBO vectors [35]. Their “SHEC” vector lacks a CP, and thus cannot form virions, and also replicates very poorly in the absence of the silencing suppressor P19. Such tobamovirus-based systems can further regulate vaccine production under highly inducible and contained conditions.

The recent discovery of adjuvant properties of TMV has sparked a renewed interest in the use of this virus as a delivery vehicle for immunotherapy. TMV particles have been demonstrated to be taken up by dendritic cells and to exhibit activation properties, resulting in robust CD8+ T cell responses [36,37]. Banik et al. used TMV particles to act as both an adjuvant and an epitope display system for vaccine development against the facultative intracellular pathogen *Francisella tularensis* that did not elicit adverse reactions when administered to mice, yet protected them against respiratory challenges with very high doses of *F. tularensis* Live Vaccine Strain LVS [38]. Similarly, Jones et al. used this virus-based delivery platform as a means to generate a malaria transmission blocking vaccine (TBV), which specifically targets proteins expressed in the mosquito midgut during *Plasmodium falciparum* development. TBV proteins, such as the Pfs25 protein, could be a potential target to reduce the transmission of malaria [39]. The TMV deconstructed vector has also been used to produce HPV vaccines based on the VLPs composed of the L1 protein. Such a vaccine could offer a safe and inexpensive vaccine for the poor in developing countries [40,41].

The MagnICON deconstructed vector is perhaps most well-known for its use in efforts to develop personalized medicine against non-Hodgkin’s lymphoma (NHL) [42]. NHL is a cancer of overproliferating B cells, with an estimated 70,000 new cases in 2014 alone. Since malignant B cells present a unique cell surface idiotype that is specific to that individual, patients can be vaccinated using their own idiotype. TMV constructs composed of the scFv subunit and full-length idiotype IgG molecules were expressed in deconstructed MagnICON vectors as heavy and light chains, which assembled into full immunoglobulins in the plant [43,44]. Each vaccine construct has successfully passed Phase I clinical trials, been demonstrated to be safe, and elicit few adverse effects. The number of patients who mounted immune responses was comparable to the results of earlier clinical trials using follicular lymphoma idiotype vaccines that have been generated using other production platforms. Furthermore, vaccine manufacture is extremely rapid, taking less than three months to obtain a completed vaccine based upon US FDA cGMP guidance from an initial biopsy [45,46]. For further information requiring the large scale production of pharmaceutical proteins using the MagnICON system, please refer to Klimyuk et al. [47].

The tobacco mosaic virus has been engineered to be tumor-specific using the tumor homing peptide cRGD, which has been functionalized to the surface of the virus. TMV particles that display cRGD can become rapidly internalized into tumor cells. Doxorubicin and other anti-cancer drugs can also be conjugated to TMV so that they can be taken up by cancer cells and released during endocytosis [48].

## 4. Vectors Based on Potexviruses

Potato Virus X (PVX), a flexuous, rod-shaped virus containing a plus-sense RNA molecule, has also been engineered extensively as an expression vector for biopharmaceuticals. The genome of PVX consists of replicase and capsid protein genes, as well as a triple gene block, whose products are responsible for virus movement. PVX has been used to express full-length proteins, fusion proteins, epitopes that are displayed on the outer surface of the assembled virus particle, and more recently, PVX nanoparticles have been demonstrated to block tumor progression in animal models [49]. For example, a PVX-based expression system has been engineered to produce the N and M proteins of Sudden Acquired Respiratory Syndrome Coronavirus (SARS-CoV) antigens [50]. The plant-derived N protein can be used to examine the presence of N-specific antibodies in the sera of patients who had been exposed to coronavirus.

PVX has been employed for the development of a universal influenza vaccine consisting of an epitope derived from the extracellular domain of H1N1 virus matrix protein 2 (M2e). The researchers fused M2e to bacterial flagellin, a strong mucosal adjuvant, in order to improve M2e immunogenicity [51]. *N. benthamiana* plants infected with a PVX vector expressing this fusion protein were able to produce 1 mg/g fresh leaf tissue or 30% total soluble protein, and inoculated mice were protected from influenza infection.

PVX has produced other antigens as well. For example, Uhde-Holzem et al. (2010) used PVX to express the epitope HVR1 from the Hepatitis C virus (HCV) as a fusion protein [52]. Mice immunized parenterally elicited an IgG response, and sera from chronically infected HCV patients reacted positively to the PVX-HVR1 epitope. Similarly, Mohammadzadeh et al. fused epitopes of the HCV core antigen to Hepatitis B virus surface antigen (HBsAg), and expressed the resulting chimeric construct from the second coat protein promoter of a PVX vector [53]. The papaya mosaic potexvirus (PapMV) has been used to express an epitope of the envelope protein of HCV to generate a long lasting humoral antibody response in mice [54]. PapMV has also been employed as a carrier for a potential universal flu vaccine, by fusing an epitope of M2e to the outer surface of PapMV nanoparticles [55].

Recently, PVX has been designed to act as a nanoparticle for tumor immunotherapy. PVX nanofilaments carrying monoclonal antibodies of herceptin (Trastuzumab) for breast cancer patients [56] could induce apoptosis in breast cancer cell lines. PVX expressing a mutant version of the HPV16 E7 oncoprotein as part of a fusion protein with lichenase protected mice from tumor growth by generating a strong cytotoxic T-cell response [57,58]. External lysine residues on the capsid protein of PVX can be functionalized to take on conjugates. Shukla et al. (2013) found that PVX accumulated in the center of solid tumors, suggesting that PVX will be useful for tissue-specific imaging and drug delivery [59].

PapMV also has immunostimulatory properties, and can act as a nanoparticle. PapMV nanoparticles can elicit an α-IFN-dependent response, and when administered intra-tumorally can slow down melanoma progression and prolong survival in animal models [58,59,60,61,62]. PapMV undergoes rapid endocytosis by antigen-presenting cells, resulting in CD8+ T cell proliferation. Mice treated systemically with PapMV nanoparticles followed by B16-OVA cells six hours later exhibited fewer tumor nodules compared to control mice. The fact that this result could not be reproduced with capsid monomers or naked virus RNA indicated that the assembled nanoparticle itself was required for immune protection [60].

## 5. Virus Expression Vectors Based on Comoviruses

The Comovirus Cowpea mosaic virus (CPMV) has undergone extensive development as an expression vector. CPMV is an icosahedral virus of 30 nm in diameter, comprised of 60 large (L) and 60 small (S) capsid proteins. The genome of CPMV is bipartite, with RNA-2 being the principal component of expression vector development. CPMV has been utilized extensively in antigenic presentation and full-length protein expression as part of a fusion protein that can undergo proteolytic cleavage to release the therapeutic protein, as well as in material science research, such as the formation of magnetic clusters and biosensors [63,64,65,66]. For example, Medicago, Inc. (Durham, NC, USA) has used the CPMV vector to generate virus-like particles (VLPs) carrying influenza virus HA antigens [67]. These VLPs protect against lethal viral challenge in animal models, and are now undergoing a Phase 2 clinical trial with over 250 volunteers. Medicago’s CPMV production system enables a vaccine to be generated within 3 weeks of the release of the influenza strain sequence information, with an easily adaptable upscaling capacity.

The CPMV deconstructed vector system pEAQ involves the expression of foreign proteins without the need for viral replication [68,69], thus relieving the cell from any hindrance created from viral cycle progression that negatively impacts protein accumulation. This involves the positioning of the foreign gene between the 5′ leader sequence and 3′ untranslated region (UTR) of RNA-2, as well as the deletion of an in-frame initiation codon found upstream of the main translation initiation site of RNA-2. The resulting vector provides for a substantial increase in foreign protein production.

A new vector, known as pCPMV-HT (Cowpea Mosaic Virus Hyper-Trans expression system) has been engineered that also provides high translational efficiency [70,71]. These vectors have been used to generate vaccines against the bluetongue virus, HIV, Dengue, and the influenza virus [72,73].

CPMV particles have also been found to bind to vimentin displayed on the surface of HeLa cells, and are able to be taken up by endocytosis [74,75,76]. The fact that CPMV can be internalized by antigen-presenting cells enables it to prime the antibody response. CPMV nanoparticles have thus been shown to directly stimulate the immune system to combat various cancers. CPMV capsid proteins can be driven to self-assemble in the absence of RNA to form empty non-infectious virus-like particles (eVLPs), which can in turn be directly applied to a tumor and alter the surrounding microenvironment to potentiate tumor immunity through the activation of quiescent neutrophils in the neighboring region. Since the immune response is highly localized, patient adverse responses are minimalized [76]. For example, Lizotte et al. used mouse models to demonstrate that CPMV nanoparticles could act as effective immunotherapies for lung melanoma and other cancers, and were capable of inducing innate immune cell-mediated anti-tumor responses [77]. The authors were also able to increase proinflammatory cytokine levels by providing CPMV nanoparticles to bone marrow cell cultures. A weekly injection with CPMV nanoparticles further reduced tumor burden in colon tumors as well as in ovarian and breast cancers. In the future, CPMV could carry anticancer drugs as a payload to improve its ability to reduce tumors.

The Cowpea mosaic virus expression vector pEAG-HT has also been used to express the extracellular domain of the rat ErbB2 in tobacco plants [78]. ErbB2 is an epidermal growth factor-related protein, and its aberrant expression can lead to a variety of cancers. Plant extracts expressing ErbB2 that were injected into mice elicited immune responses and potent antitumour activity.

## 6. Merging of Expression Systems Using Two Different Plant Viruses

One possible direction of vector development may include the merging of genetic elements from two vastly different virus expression systems. This next section describes two such systems.

In the first example, Mardanova et al. utilized an expression vector construct based on PVX, along with a translational enhancer from RNA 4 of the alfalfa mosaic virus (AMV), and combined it with the CPMV-based pEAQ-HT vector backbone driving a suppressor of gene silencing to create a new vector expression system (pEff) [79]. pEff is smaller in size and has been used to express a candidate influenza vaccine protein m2e fused to HBc in the form of virus-like particles to levels several times higher than those either PVX or the CPMV-based vector system has produced alone.

In the second example, TMV has been used to express the genome of the Alphavirus Flock house virus (FHV), a small insect virus that can replicate to high levels in plants without inducing apoptosis. By including origins of assembly (ori) from TMV into the Flock house virus genome, a pseudovirus consisting of the FHV genome encapsidated by TMV coat protein was generated. Since TMV behaves like an adjuvant and can stimulate an immune response, this pseudovirus approach can be used to produce novel vaccines from plants [80].

## 7. Vectors Based on Geminiviruses

Geminiviruses, with their twinned capsid morphology, are single-stranded circular DNA viruses, and are capable of undergoing replication in a broad range of host plants at a high copy number. As a result, geminiviruses are suitable to develop as expression vectors [81]. The geminivirus Bean yellow dwarf virus (BeYMV) has been engineered to produce efficacious monoclonal antibodies to the Ebola, Zika, and West Nile viruses, respectively [82]. Edward Rybicki’s group in South Africa has utilized BeYDV to express constructs containing HPV L1 and L2 capsid proteins along with a secreted embryonic alkaline phosphatase (SEAP) reporter construct to assemble into the encapsidated HPV pseudovirions (PsV) in tobacco plants [83,84].

A geminivirus vector based on the Beet Curly top virus (BCTV) has been used to generate a vaccine against hepatitis A [85]. As another example, the dual-module in-plant activation (INPACT) expression platform was developed in tobacco yellow dwarf virus (TYDV) to control foreign gene expression [86,87]. By interrupting an expression cassette by a plant intron, the protein of interest is expressed only after post-transcriptional processing, thus offering further control of expression of the gene of interest.

## 8. Impact for Developing Countries

Fundamental difficulties remain for populations of remote, impoverished regions to access vaccines and other biopharmaceutical protein, including high cost and limited accessibility. This prevents many from achieving better health, and as a result better livelihoods for themselves and their families. Plant-made vaccines could slow the spread of infectious diseases that are major killers, such as HIV, HBV, and malaria. Plants possess other advantages as expression systems; they do not harbor mammalian pathogens and in certain instances can undergo similar post-translational modifications to their mammalian counterparts. Pharmaceuticals derived from plants can easily be purified, or in specific cases merely partially purified prior to oral administration.

Plant-made biopharmaceuticals to fight cancer are increasing in number. Plant-made vaccines which combat oncogenic viruses that are of concern to resource poor countries, such as Hepatitis B, Hepatitis C, and Human papillomavirus, are under development and could make a huge impact on the quality of life for hundreds of millions of people. The generation of monoclonal antibodies to combat cancer in a variety of plants could also assist developing countries. The recent discovery of plant virus nanoparticles to target and penetrate solid tumors is particularly interesting [88,89,90,91]. Plant virus nanoparticles with drugs functionalized to their surfaces would be efficacious, low in cost, easy to produce en masse, and low in toxicity, which are ideal qualities for a resource-poor setting.

Plant virus particles can also be a platform technology for developing nations to utilize for growing their own pharmaceutical industries. By equipping poor countries with virus constructs and freedom to operate, a plant-made pharmaceutical industry could become a reality. Improvements in manufacturing infrastructure, financial support, and the maturation of a regulatory framework surrounding plant-derived pharmaceuticals are all essential for their commercialization in the developing world. If these obstacles are overcome, it would seem that many of the examples presented in this review could play a significant role in improving the lives of many.
